# Pattern of childhood ocular morbidity in rural eye hospital, Central Ethiopia

**DOI:** 10.1186/1471-2415-14-50

**Published:** 2014-04-15

**Authors:** Zelalem Addisu Mehari

**Affiliations:** 1 Nigist Eleni Mohammed Memorial Hospital, Eye unit, P.O. Box 672, Hossana, Ethiopia

**Keywords:** Childhood, Amblyopia, Refractive errors, Ocular morbidity, Ethiopia

## Abstract

**Background:**

This study was aimed to determine the pattern of childhood eye disorders in patients attending outpatient eye department of a rural eye hospital in central Ethiopia.

**Methods:**

A cross-sectional survey of ocular morbidity among children less than 15 years of age who presented at a rural eye hospital in central Ethiopia between August – October 2012 was conducted. Demographic data, visual acuity, source and type of injury, type of refractive errors and diagnosis were collected and analyzed using SPSS. A *p* value less than 0.05 was considered statistically significant.

**Result:**

A total of 735 children were examined in this study. The age range of the children varied from three months to 15 years of age. The mean (SD) age of the study population was 9.37 (4.95) years. 369 (50.2%) of the patients were females. The majority of cases were observed in older children (11–15 years) accounting for almost half of all the cases. The most common ocular morbidity encountered was conjunctivitis (35%), then ocular trauma (11.8%), refractive error (11.4%) and trachoma (7.6%). Bilateral visual impairment (UCVA < 6/18 in the better eye) was found in 119 children, and the causes were refractive errors (47.1%), keratitis/corneal opacity (16%), amblyopia (14.3%), ocular trauma (11.8%), cataract (6.3%), Glaucoma (2%) and uveitis (2%).

**Conclusion:**

The three most common causes of childhood ocular morbidity in this study were conjunctivitis, ocular injuries and refractive errors. These disorders require attention of all the health professionals for proper management or early referral because they can lead to visual impairment and blindness. Health education is necessary for the prevention of childhood eye injuries, as well as early presentation of children to eye care centers for the treatment of eye disorders.

## Background

Childhood ophthalmic disorders can seriously impact on development, education, future employment opportunities and quality of life. The consequences are especially severe in low resource settings where resources and education are lacking. Poor education and an inability to fully participate in daily life greatly add to the difficulty and suffering those children with poor vision or blindness experience. Eye diseases in children are an important reason for medical consultation [1] and children should receive prompt and proper eye care to avoid vision problems and eye morbidities [2]. Specifically, addressing childhood blindness is a priority because these individuals are blind for several decades. Data on the prevalence and causes of blindness and severe visual impairment in children are required to appropriately plan and evaluate preventive and curative services, including special education and low vision services.

Pediatric ophthalmic disorders can arise because of events that occur during the prenatal or neonatal periods or childhood. Optical, orthoptic, medical and surgical interventions can be employed to manage pediatric ophthalmic disorders. These should be selected carefully in children, who have unique problems in terms of ocular morbidities, due to their inability to articulate their problems, and because of the potential to develop amblyopia in the event of visual impairment [3]. Globally, an estimated 70 million blind years are caused by childhood blindness. Approximately 500,000 children become blind every year, which is equivalent to one child every minute; 60% die within 1 to2 years of becoming blind [4]. The prevalence of childhood blindness is especially high in low-resource areas; among 1.5 million blind children worldwide, 70-90% of them are in the poorest countries of Africa and Asia [5].

Various ocular morbidity surveys have estimated the magnitude of eye diseases among children. In a survey conducted in Nigeria, refractive errors (25.7%), vernal conjunctivitis (25.3%), eye injuries (13.3%), and corneal inflammation (12.5%) were the leading causes of childhood eye morbidity [6]. In a study in Tikrit, Iraq, allergic conjunctivitis (27%), refractive error (14.6%), ocular trauma (13.8%),infection (12.7%), squint (12.1%) and nasolacrimal duct obstruction (NLDO, 5.2%) were the most common conditions treated in an outpatient department [7]. In a population based study done in central Ethiopia, it is shown that 51.6% of children under 10 years of age suffer from active trachoma [8]. School based ophthalmic screening for ocular abnormalities and low vision in school children of the same town revealed one or more ocular abnormalities in 62% of the students and refractive error was also found to be the leading cause of low vision in this study [9].

Unfortunately there is inadequate data on causes and prevalence of ocular morbidities amongst children in Ethiopia. The aim of this study was to determine the pattern of childhood eye disorders across age groups in children attending an outpatient eye department in a rural eye hospital in central Ethiopia.

## Methods

A hospital based descriptive cross-sectional study was done in the outpatient eye department of rural eye hospital (Grarbet Eye Hospital) in central Ethiopia. The eye hospital serves a population of about 1.5 million residing in six woredas (sub-districts) of the Silti and Gurage zones of southern regional state. The study took place from 1^st^ August to the end of October 2012. All consecutive children with ocular disease seen in the eye unit for the first time were included in the survey. In this study 735 children were recruited up to the age of 15 years. Children above 15 years of age, repeated cases (cases within the study) and those presented for a medical check-up and had no ocular diseases were excluded from the study.

Demographic data, visual acuity, source and type of ocular disorder and the type of refractive error were included in the questionnaire. Ocular disorders were divided on anatomical basis as disorders affecting conjunctiva, cornea, sclera, lens, uvea, retina, optic nerve, ocular muscles, nasolacrimal duct system, lids, orbit and refractive system. Visual acuity was measured at a distance of six metres using the Snellen E chart on presentation and was categorized as WHO classifications; ≥6/18, <6/18-6/24, <6/24-6/60, <6/60-3/60 and <3/60-NLP. Those children whose age is greater than 5 years and had decreased visual acuity not attributable to another cause at presentation, and all less than 5 years old underwent refraction.

Children were examined with the help of their parents or guardians by Ophthalmologist or cataract surgeon. Anterior segment examination was done with slit lamp and torch. Posterior segment examination was performed after dilating pupil using direct and indirect ophthalmoscope and fundus camera. Intraocular pressure was checked with air puff non-contact tonometer. If the child had decreased vision the examiner sent him/her to vision centre/refraction clinic/ for refraction, the experienced optometrist undertook objective and subjective refraction and then rechecked with correction to confirm RE is the cause for the visual impairment.

Strabismus assessment was done using an occluder (cover uncover test). We only report one main diagnosis for each patient. Main diagnosis of Patients in the study represent the diagnosis, condition, problem or other reason for the encounter/visit that is ***chiefly responsible*** for the outpatient services provided. The main diagnosis of bilaterally visual impaired children was taken as causes of bilateral visual impairment. It is unfortunate that all the 47 cases main reason for the outpatient service was trachoma. But active trachoma was not always chosen as main diagnosis in all cases.

The patients' age was stratified into three age groups(<5 years,6-10 years and11-15 years).The type of injury was classified according to Birmingham’s Eye Trauma Terminology classification(BETTS) [10] as closed globe injuries for contusions, lamellar lacerations and superficial foreign body while ruptures, penetrating, perforating and intraocular foreign body laceration as open globe injury.

### Definitions and analysis

Myopia was defined as a spherical equivalent of −0.50 DS (dioptre sphere) or greater in one or both eyes. Hyperopia was defined as a spherical equivalent of +2.00DS or more in one or both eyes. A cylindrical power of −0.50 DC (D cylinder) or greater was considered as astigmatism. For comparison with other studies, those that met criteria more than one category of refractive error were classified as astigmatism (simple/compound myopic or hyperopic astigmastism, or mixed astigmatism).

Amblyopia is defined as a difference of two lines or more in best corrected vision (on an eye-chart test of visual acuity) between the two eyes or a best corrected vision of 6/12 or worse in the affected eye.

Strabismic ambylopia is suppression of central vision resulting from deviation of the eye that is not straight.

Deprivation ambylopia is suppression of central vision when cataracts or similar condition deprive children’s eyes of visual experience.

Refractive ambylopia happens when there is large or unequal amount (at least 2 Ds) of refractive error in child’s eyes (anisometropic refractive amlyopia). If both eyes are out of focus and not found and corrected in early age, both may become ambylopia (Bilateral refractive ambylopia).

Corneal visual impairment encompasses a wide variety of infectious and inflammatory eye diseases that cause scarring of the cornea, the clear membrane that covers the outside of the eye. Significant scarring ultimately leads to functional vision loss.

Statistical analysis was performed with Statistical Package for the Social Sciences (SPSS) version 12. Proportions were used to present the results. P-value and confidence intervals were also used to indicate level of significance of the findings. The study adhered to the tenets of the Declaration of Helsinki and was conducted after obtaining ethical approval from the Institution Board of the hospital.

## Results

**Figure 1:**
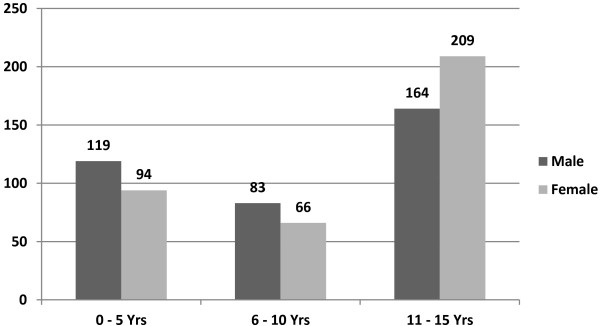
Age and sex distribution of children.

A total of 735 children between 3 month and 15 years of age were examined in the eye unit during the study period. The gender distribution of the patients was 366 (49.8%) males and 369 (50.2%) females. The mean (SD) age of the study population was 9.37 (4.95) years. The age group with the most patients was the 11–15 year age group, followed by the ≤ 5 years age group (Figure 1).

Visual acuity data were analyzed monocularly and binocularly, and bilateral visual impairment (UCVA <6/18 in the better eye) was found in 119 children (Table 1). The cause of visual impairment were refractive errors (47.1%), keratitis/corneal opacity (16%), amblyopia (14.3%), ocular trauma (11.8%), cataract (6.8%), glaucoma (2%) and uveitis (2%).

**Table 1:** Visual impairment (VA < 6/18 in the better eye) distribution by gender and Age (n = 735)

**Sex**	**Age group (Yr)**	≥ **6/18**	**<6/18-6/60**	**<6/60-3/60**	**<3/60-NLP**	**Indeterminable**	**Total no (%)**
Male	≤ 5	4	1			107	112 (15.2)
	6-10	63	5	4	1	10	83 (11.3)
	11-15	135	20	13	3		171 (23.3)
Female	≤ 5	1				93	94 (12.8)
	6-10	48	11	3	2	2	66 (9.0)
	11-15	153	33	17	6		209 (28.4)
Total		404 (55.0)	70 (9.5)	37 (5.0)	12 (1.7)	212 (28.8)	735 (100)

The proportion of various ocular diseases is shown in Table 2. Conjunctivitis was the most common ocular disorder seen in 35% of the study subjects, and allergic conjunctivitis was accounted the largest part of these cases (72%). Ocular trauma accounted for 11.8%, of these patients 74% (n = 64) were males and 26% (n = 23) females. Refractive errors accounted for 11.4% with female preponderance (54%). Keratitis (of any cause) was seen in 77children; male preponderance 61%, and higher incidence noted among older children 11–15 years constitute 52%. Trachoma was found in 56 children (7.6%), of these active trachoma was seen in 47 children and 9 cases of trachomatous trichiasis were also identified. Amblyopia constitutes 3.1% of the cases, of these 52% with anisometropic amblyopia, 35% refractive amblyopia and 13% with strabismic amblyopia. Tumors accounted for 1.2% of the childhood eye diseases during the study period, with three cases (33.3%) of dermoids, two cases (22.2%) each of retinoblastoma and lymphoma and one case (9.1%) each of a conjunctival mass and an eye lid mass.

**Table 2:** Distribution of childhood eye disease by gender (n = 735)

**Type of ocular morbidity**				
	**Male**	**Female**	**Total no %**	
Conjunctivitis	131	127	258	35.1
Keratitis	30	47	77	10.5
Active trachoma	24	23	47	6.4
Stye/Chalazion/Blephritis	18	28	46	6.3
TT	4	5	9	1.2
Cornea opacity	9	4	13	1.8
RE	39	45	84	11.4
Ocular trauma	64	23	87	11.8
Cataract	14	6	20	2.7
Amblyopia	9	14	23	3.1
Strabismus	5	8	13	1.8
Tumor	3	6	9	1.2
Uveitis	2	6	8	1.1
NLDO	5	10	15	2
VAD	2	3	5	0.7
Glaucoma	3	0	3	0.4
Others	4	14	18	2.5

TT- trachomatous trichiasis, VAD-vitamin A deficiency, NLDO-nasolacrimal duct obstruction, CO- corneal opacity, RE-refractive error.

Ocular injuries were the second most common ocular disorders seen in 87 (11.8%) children, male preponderance (74%) and higher proportion noted among school age children (77%) of all traumatic eye disorders. Most number of trauma occurred at home (55%, n = 48) followed by street and highway (35.6% n = 31), school (9.2%, n = 8). The commonest type of injury was closed globe injury, accounting for 61% (n = 53) of the total. Corneal and/or sclera lacerations occurred in 36.8%, Contusion in 23% and superficial foreign body in 11.5% of eyes (Table 3).

**Table 3:** The relationship between the types of injury with gender and age (n = 87)

**Type of ocular injury**	**Sex**		**Age (years)**			**Total no of patients (%)**
	**M**	**F**	**0 – 5**	**6 – 10**	**11 – 15**	
Contusion	12	8	4	8	8	20 (23)
Superficial FB	8	2	2	5	3	10 (11.5)
Adenxal/lacrimal Laceration	6	2	2	2	4	8 (9.2)
Corneal Burn	0	2	0	1	1	2 (2.3)
Partial thickness wound	10	2	3	4	5	12 (13.8)
Rupture	1	0	0	0	1	1 (1.1)
Penetration						
Corneal	15	3	4	5	9	18 (20.7)
Scleral	3	2	0	2	3	5 (5.8)
Corneo-scleral	7	2	1	3	5	9 (10.3)
Perforating	1	0	0	0	1	1 (1.1)
IOFB	1	0	0	1	0	1 (1.1)
Total	64 (73.6%)	23 (26.4%)	20 (23%)	27 (31%)	40 (46%)	87 (100)

FB- Foreign body, IOFB-Intra ocular foreign body.

Of the 735 children, 84 children were diagnosed with a refractive error based on retinoscopy refraction, constituting an overall proportion of 11.4% (84/735). The classification of refractive errors was shown according to the following distribution: astigmatism was the most frequent type of refractive errors (53.6%); followed by myopia 40.5% and finally hypermetropia with 5.9% (Table 4).

**Table 4:** Type and distribution of refractive errors by age group (n = 84)

**Age (yrs)**	**Myopia**	**Hyperopia**	**Astigmatism**	**Total**
	**No. (%)**	**No. (%)**	**No. (%)**	**No. (%)**
0 – 5	1 (1.2)	0	0	1 (1.2)
6 – 10	5 (5.9)	1 (1.2)	8 (9.5)	14 (16.7)
11 – 15	28 (33.3)	4 (4.8)	37 (44.0)	69 (82.1)
Total	34 (40.5)	5 (5.9)	45 (53.6)	84 (100)

## Discussion

The limitations of clinic - and hospital - based surveys are well recognized. The patients who seek help are self selecting and not necessarily representative of the total regional population with eye disease . Unfortunately, diseases in children are often difficult to diagnose and treat because the children may be unwilling or unable to cooperate in a complete examination and treatment regimen. In this study, pediatric ophthalmic disorders accounted for 18.6% of all new patients seen in the eye unit during the study period. The proportions of males and females were similar, and around 71% of the patients were school aged.

Similar to this study, previous reports described that conjunctivitis was the most common ocular surface disorder in children [11-14]. This is likely due to the dusty local environment (largely farming communities), the study season (which favors vernal catarrh), and the climate itself .Moreover, rural living is a risk factor for the development of chronic allergic conjunctivitis in children [15]. Both genders were affected roughly equally in this study, which differed from other reports of male prevalence [16]. The higher proportion noted among older children may be related to factors affecting late presentation for eye- care utilization for a chronic recurrent disease rather than actual disease prevalence in this age group.

Ocular trauma was the second-most common ocular morbidity seen in this study, which accounted for 11.8% of the all ocular morbidity observed during the study period. The proportion of ocular injuries is higher in developing countries and consists of largely preventable causes of monocular visual impairment and blindness. Hospital-based studies reported that 5% to 16% of all ophthalmic admissions to eye hospitals/units are related to ocular injuries [17]. Approximately 75% of the ocular injuries occurred in male in this study, which was equivalent to trends reported by other authors [18,19]. Other studies performed in this eye unit described that patients with ocular injury account for 5% of all new patients overall, and ocular injury in the pediatrics group accounted for 29.7% of the total ocular injuries treated in the eye unit during the study period. Seasonal variation and distance travelled by the patients to get ophthalmic services were the factors that reduced the number of ocular injuries observed during the study period in the eye unit in the previous report [19]. Similar to the study done in Malawi [20], in this study ocular injury at home still accounts for a high percentage of injuries treated during the study period. In most studies, blunt objects, such as sticks or stones, were the main cause of eye trauma [19,21]. Findings from this study concur with previous studies in the region.

Refractive errors which account mostly for low vision and visual impairment is also the third largest cause of pediatric ocular morbidity in this study, and accounted for 11.4% which is similar with the study carried out in, Nigeria 14.3% and Iraq 14.6% [7,22]. Astigmatism was the most common type of refractive error and accounted for 53.6% of refractive error in this study. This is similar to a study carried out in Pakistan where astigmatism was the most common and accounted for 46.25% [23]. The study done among schoolchildren in six districts in the study area revealed overall rate of myopia in students 7-15 years of age as 6.0%, astigmatism 2.1% and hyperopia 0.33% [24]. The higher proportion of refractive error reported in older children could be due to better articulation by older children and detection of their visual problems by their families or teachers.

Keratitis of any cause contributed 10.5% of ocular morbidity in the study, and is particularly recognized for causing blinding corneal scars. Children presenting with keratitis are at risk of developing irreversible ocular deficits, such as those resulting from amblyopia [25]. Therefore, the diagnosis and treatment of microbial keratitis in children is of utmost importance. The frequency of keratitis in this study was consistent with the report in south-western Nigeria in which corneal infection constituted more than one third of the infections of the eye and adenxia [22]. Corneal scarring was also one of the ocular morbidity seen in the study groups, and leads to visual impairment that is difficult to treat except with corneal surgery, which may not be easily available to the population; then, in addition, it is even more problematic in childhood because amblyopia may develop, which can then limit the benefits of surgery later in life.

The national survey on blindness, low vision and trachoma in Ethiopia revealed that prevalence of active trachoma in 1–9 years old children was very high and clearly indicates the burden of trachoma in a rapidly growing population [26]. Moreover, the southern regional state has one of the highest prevalence of active trachoma in the survey (33.2%). The present study was carried out in a population where trachoma had been prevalent and mass treatment of Zithromax has been distributed for the last three years and intensive outreach programs have been conducted targeting implementation of SAFE (S = surgical care, A = antibiotics, F = facial cleanliness, E = environmental improvement) strategy in the surrounding communities. Even if the result of this study encourages community education as an essential part in prevention of trachoma, population based prevalence survey is mandatory to evaluate the outcome of trachoma control program carried out in study area.

The proportion of strabismus in this study (1.8%) was almost similar to the 2.4% in Nigeria [22] study, but much lower than the 12% in Iraq [7].

Despite some limitations, the results of this study give useful information on the epidemiology of pediatric ophthalmic disorder in rural central Ethiopia. Future population-based surveys and prospective clinical studies focussing on pediatric ocular morbidities should be performed.

## Conclusions

In conclusion, it is a difficult task to detect and treat children with ocular morbidity because they are unable to articulate their problems and are sometimes are not cooperative for examination and treatment. However, it is important to approach children with the same pattern with which one would approach an adult. The most common causes of childhood ocular morbidity in this study were conjunctivitis, ocular injuries and refractive errors. These disorders require attention of all health professionals for complete management or early referral because they lead to visual impairment and blindness. Health education is necessary for the prevention of childhood eye injuries, as well as early presentation of children to eye care centers for the treatment of eye disorders.

## Competing interests

The author declare that he has no competing interest.

## Pre-publication history

The pre-publication history for this paper can be accessed here:

http://www.biomedcentral.com/1471-2415/14/50/prepub
